# Supplementary material for: The Clinical Impact of Hepatic Arterial Infusion Chemotherapy New-FP for Hepatocellular Carcinoma with Preserved Liver Function
Source: Cancers (Basel). 2022 Oct 5;14(19):4873. doi: 10.3390/cancers14194873 (PMC9562659; doi:10.3390/cancers14194873)
Supplement: Supplementary file 1 [file cancers-14-04873-s001.zip › cancers-1896612-supplementary.pdf]

Supplementary Table S1

Patient characteristics before and after propensity score matching in cohort 1

	Before matching n=342			After matching n=76		
Patient characteristics	New FP n=51	Sorafenib n=291	*p*-value	New FP n=38	Sorafenib n=38	*p*-value
Age (years)	69.4±10.8	72.3±8.48	0.7870	69.2 ±11.5	68.1±9.7	0.4385
Sex Male / Female	42/9	235/56	0.0315	29/9	34/4	0.2223
HCV/HBV/non-viral	14/18/19	38/187/66	<0.0001	11/15/12	10/15/13	0.7975
Child-Pugh score (5/6)	27/24	193/98	0.1551	20/18	21/17	0.8180
Tumor characteristics						
Tumor size	6.55±4.31	3.35±2.44	<0.0001	4.85 ±2.57	4.81 ±3.05	0.6929
AFP (ng/ml)	6,415.0± 27,282.7	2,255.7± 10,174.2	0.1044	1,192.61±50 7.85	1,616.81± 589.07	0.2488
DCP (mAU/ml)	19,621.9± 50,771.0	7,759.5± 44,644.0	0.1528	6,989.92±2, 401.02	2,578.28±1, 244.67	0.3086

EHS: extrahepatic spread, AFP: alpha-fetoprotein, DCP: des-gamma carboxyprothrombin

Supplementary Table S2

Patient characteristics before and after propensity score matching in cohort 2

	Before matching n=404			After matching n=156		
Patient characteristics	New FP n=289	Sorafenib n=115	*p*-value	New FP n=78	Sorafenib n=78	*p*-value
Age (years)	69.2±10.4	67.2±10.1	0.6270	69.0 ±10.5	69.1±9.3	0.9802
Sex Male / Female	225/64	90/25	0.9291	57/21	61/17	0.4556
HCV/HBV/non-viral	46/133/110	32/52/31	0.9122	20/39/19	16/41/21	0.4472
Child-Pugh score (5/6)	179/110	66/49	0.4367	48/30	48/30	1.0000
Tumor characteristics						
Tumor size	8.29±4.61	5.62±3.80	<0.0001	6.39 ±3.70	6.38 ±4.03	0.6767
AFP (ng/ml)	33,462.96 ± 10,730.00	22,381.79 ± 7,472.93	0.5425	38,863.41±25,463.26	19,476.54±9,537.71	0.4200
DCP (mAU/ml)	21,031.09 ± 3,678.45	19,673.54 ± 4,727.73	0.8362	23,517.80±6,916.51	23,788.93±6,576.50	0.9774

EHS: extrahepatic spread, AFP: alpha-fetoprotein, DCP: des-gamma carboxyprothrombin

Supplementary Table S3

Patient characteristics before and after propensity score matching in cohort 3

	Before matching n=315			After matching n=12		
Patient characteristics	New FP n=9	Sorafenib n=306	*p*-value	New FP n=6	Sorafenib n=6	*p*-value
Age (years)	63.6±16.3	70.4±9.1	0.0408	67.0 ±5.8	70.1±3.2	0.6047
Sex Male / Female	5/4	241/65	0.1216	4/2	4/2	1.0000
HCV/HBV/non-viral	2/5/2	49/150/107	0.6182	1/4/1	1/4/1	1.0000
Child-Pugh score (5/6)	6/3	205/101	0.9848	4/2	4/2	1.0000
Tumor characteristics						
Tumor size	7.45±3.27	2.39±2.83	<0.0001	5.58 ±1.84	4.21 ±2.10	0.2674
AFP (ng/ml)	11,848.5± 7,635.0	15,907.1± 5,709.6	0.9028	16,612.33±11,192.84	4,817.28± 3,125.62	0.3703
DCP (mAU/ml)	20,995.1± 13,163.2	10,988.50 ± 2,239.7	0.4931	27,858.50±16,904.60	1,633.76±666.98	0.7586

EHS: extrahepatic spread, AFP: alpha-fetoprotein, DCP: des-gamma carboxyprothrombin

Supplementary Table S4

Patient characteristics before and after propensity score matching in cohort 4

	Before matching n=175			After matching n=76		
Patient characteristics	New FP n=67	Sorafenib n=108	*p*-value	New FP n=38	Sorafenib n=38	*p*-value
Age (years)	65.7±12.2	66.0±10.9	0.8719	65.7±12.0	65.6±11.0	0.9840
Sex Male / Female	53/14	92/16	0.2995	31/7	31/7	1.0000
HCV/HBV/non-viral	14/24/29	31/43/34	0.5973	37/19/22	10/15/13	0.7910
Child-Pugh score (5/6)	38/29	52/56	0.1579	20/18	18/20	0.6463
Tumor characteristics						
Tumor size	9.77±4.56	6.06±4.46	<0.0001	8.11±3.42	8.39 ±4.76	0.7865
AFP (ng/ml)	22,015.90 ± 8,011.78	62,671.88 ± 23,228.24	0.2403	22,838.03±12,327.88	31,007.09±18,769.13	0.9206
DCP (mAU/ml)	37,267.56 ± 13,735.02	37,973.16 ± 15,856.06	0.9753	22,939.05±11,315.75	19,218.84±6,024.81	0.9158

EHS: extrahepatic spread, AFP: alpha-fetoprotein, DCP: des-gamma carboxyprothrombin

Supplementary Table S5

Patient characteristics before and after propensity score matching in cohort 5

	Before matching n=291			After matching n=156		
Patient characteristics	New FP n=177	Sorafenib n=114	*p*-value	New FP n=78	Sorafenib n=78	*p*-value
Age (years)	67.0 ± 11.3	65.2 ± 11.1	0.1893	66.6 ±10.9	65.3±10.7	0.4581
Sex Male / Female	138/39	89/25	0.3908	62/16	60/18	0.6981
HCV/HBV/non-viral	71/37/69	40/40/34	0.4237	37/19/22	27/28/23	0.8597
Tumor characteristics						
Tumor size	9.45 ±5.09	6.44 ±4.64	<0.0001	7.62 ±4.21	7.26 ±4.75	0.6184
Vp4 (+/－)	67/ 110	37/ 77	<0.4014	21/57	26/52	0.3829
EHS (+/－)	50/ 63	496 / 484	<0.0001	24/54	21/57	0.5960
AFP (ng/ml)	42,524.2± 16,973.0	71,577.18 ± 243,517.19	0.3559	48,645.45± 248,475.48	52,073.81± 163,296.82	0.9184
DCP (mAU/ml)	31,275.6± 6,015.9	43,862.82 ± 163,073.45	0.0365	30,379.13± 95,631.52	27,714.78± 61,755.77	0.8360

Vp4: tumor invasion into the trunk of portal vein, EHS: extrahepatic spread, AFP: alpha-fetoprotein, DCP: des-gamma carboxyprothrombin
